# Acupuncture Ameliorates Depressive Behaviors by Modulating the Expression of Hippocampal Iba-1 and HMGB1 in Rats Exposed to Chronic Restraint Stress

**DOI:** 10.3389/fpsyt.2022.903004

**Published:** 2022-06-06

**Authors:** Lu Chen, Huili Jiang, Tuya Bao, Yu Wang, Hong Meng, Yang Sun, Pengfei Liu, Songxiao Quan, Wenshan Li, Simin Qi, Xiujun Ren

**Affiliations:** ^1^School of Acupuncture-Moxibustion and Tuina, Beijing University of Chinese Medicine, Beijing, China; ^2^Research Center of Mental and Neurological Disorders, School of Acupuncture-Moxibustion and Tuina, Beijing University of Chinese Medicine, Beijing, China; ^3^Institute of Acupuncture and Moxibustion, China Academy of Chinese Medical Sciences, Beijing, China; ^4^School of Science, Beijing Technology and Business University, Beijing, China

**Keywords:** acupuncture, depression, microglia, HMGB1, neuroinflammation

## Abstract

The antidepressant mechanism of acupuncture has not been fully elucidated recently. Thus, the objective of the present study is to investigate the antidepressant mechanism of acupuncture of modulating the neuroinflammation induced by high mobility group box-1 (HMGB1) in rats subjected to chronic restraint stress (CRS). Forty-four male Sprague Dawley rats were randomly divided into control, model, escitalopram, and acupuncture group. Except for rats in the control group, all rats were exposed to CRS for 21 days continuously. Rats in the escitalopram group were subjected to a suspension of escitalopram and saline. One hour before CRS procedures, acupuncture was performed at Baihui (GV20) and Yintang (GV29) for rats in the acupuncture group, 20 min per day for 21 days. All rats in each group were conducted to detect the body weight, sucrose preference test at 0, 7, 14, 21 days to evaluate the depression-like behaviors. The expression of microglial activation and HMGB1 in the hippocampus was detected by immunofluorescence. The expression of hippocampal interleukin-10 (IL-10) was detected by western blot. And the content of serum tumor necrosis factor-α (TNF-α) was detected by the enzyme-linked immunosorbent assay method. CRS-exposed rats showed obviously decreased body weight and sucrose preference when compared with the control group, which was reversed by acupuncture. The results have also shown that acupuncture ameliorated the CRS-induced activation of microglia and HMGB1 in the hippocampus CA1 region. Furthermore, acupuncture reduced the stress-induced upregulation of TNF-α in serum. Collectively, the current study highlights the role of acupuncture in alleviating depressive behavior associated with stress-induced neuroinflammation mediated by HMGB1 in the CRS model of depression.

## Introduction

Depression is one of the most common mental disorder diseases, which is also a leading cause of global burden (1, 2). Depression often presents with durable and significant sadness, lack of interest and pleasure, or appetite and sleep disorders, cognitive bias and cognitive impairment (1, 3). Reports have showed that approximately 5% of adults worldwide suffer from depression and even more than 700, 000 people are subjected to suicide every year. Depression is not only the major contributor to the global disease burden, but also the main cause of disability all over the world (4). At present, the treatment for depression is mainly pharmacological antidepressants, which has been confirmed the rate of non-responsiveness ranging from 30 to 50% (5). The antidepressants are also likely to cause side effects such as headache, insomnia, tachycardia, hypertension, anorexia, or gastrointestinal reactions, which might bring seriously side effects to the depressed patients (6, 7). Currently, the delayed curative effect, and insufficient incidence and remission rate indicate that multi-factors are involved in the etiology of depression (8). Accordingly, there is urgent challenge for us to illustrate the pathological mechanism of depression and explore continuously more comprehensive antidepressant strategies.

Currently, the pathogenesis of depression is considered to be related to heredity and social environment (1, 9). Monoamine neurotransmitter disorder, neuroendocrine changes, neuroplastic injury, or gut microbiota impairment have been investigated to be involved in the etiopathogenesis of depression. However, the pathophysiologic cause of depression is still unknown. And no clinically biological diagnostic markers or biological screening tests are currently available. Notably, studies have shown that the occurrence of depression is closely related to the over-activation of the immune system and the increased secretion of cytokines (5, 10). The pro-inflammatory state has been proved to be an important link in the pathological process of depression (11–13). And it has reported that the inflammatory response is positively correlated with the severity of depression (14). Studies have shown that the stress-induced neuro-inflammation is the key pathological process of depression (2, 14, 15). It has been identified that the activation of microglia is a specific manifestation of the neuroinflammation of the central nervous system (CNS) mediated by stress (2, 12, 14). Studies have indicated that hyperactivation of microglia can induce depression by secreting proinflammatory factors, which might trigger inflammatory response and inhibit neurogenesis in hippocampus (15, 16). In addition, research has shown that insufficient expression of microglia can also induce the degeneration of hippocampus and contribute to depression (17). It has been reported that the stress-mediated activation of microglia could contribute to depression-like behaviors and neuroinflammation in hippocampus. Besides, the increase of inflammatory mediators, including IL-6, IL-18, IL-1 and IL-4, were also observed in the hippocampus of rats exposed to chronic stress (18). Meanwhile, data from the investigation have confirmed that the inhibition of the hippocampal microglia activation and neuroinflammation alleviated depression-like behaviors (16, 19). Hence, the microglia activity at homeostasis may provide a way to target therapy for depression.

High mobility group box-1 (HMGB1), located outside the cell, is considered to be the endogenous risk factor and initiation signal of neuroinflammation (20, 21). Findings from the animal experiment have indicated that activation of HMGB1 could promote depressive-like behaviors in stress models of depression (22, 23). Exposure to chronic unpredictable stress (CUS) has been found to induce depressive-like behaviors, which might be mediated by activating HMGB1-RAGE pathway and upregulation of HMGB1 mRNA in enriched hippocampal microglia (24, 25). It has also been identified the significantly upregulated expression of HMGB1 in the serum and cerebral cortex of chronic unpredictable mild stress (CUMS)-induced depression model (26). HMGB1 has been considered to be the mediator to initiate the microglia sensitization and activation of proinflammatory cytokines (20, 27, 28).

Acupuncture, one part of traditional Chinese medicine with a history of more than 3,000 years, has become one of the treatment methods for depression recommended by the WHO (29, 30). Currently, data from various studies have verified the safety and effectiveness of acupuncture in the treatment of depression by alleviating stress-induced depressive-like behaviors of animal model of depression (31, 32), and improving the symptoms and quality of life of patients with depression (30, 33). It has demonstrated that the antidepressant effect of acupuncture is mainly through increasing hippocampal and network neuroplasticity, and inhibiting the stress-induced inflammation and apoptosis (32, 33). However, whether acupuncture exerts antidepressant effect by regulating the neuroinflammation mediated by hippocampal Iba-1 and HMGB1 has not been fully elucidated.

Therefore, our present study established the depression rat model of CRS and aimed to investigate the antidepressant mechanism of acupuncture of modulating the neuroinflammation induced by HMGB1. The expression of microglial activation and HMGB1 in the hippocampus was detected by immunofluorescence. The expression of hippocampal interleukin-10 was detected by western blot. And the content of serum tumor necrosis factor-α (TNF-α) was detected by enzyme-linked immunosorbent assay (ELISA) method. We aimed to elucidate the potential mechanisms underlying the antidepressant effect of acupuncture, which might provide new experimental evidence for new therapies in the treatment of depression.

## Materials and Methods

### Animals

**Figure 1:**
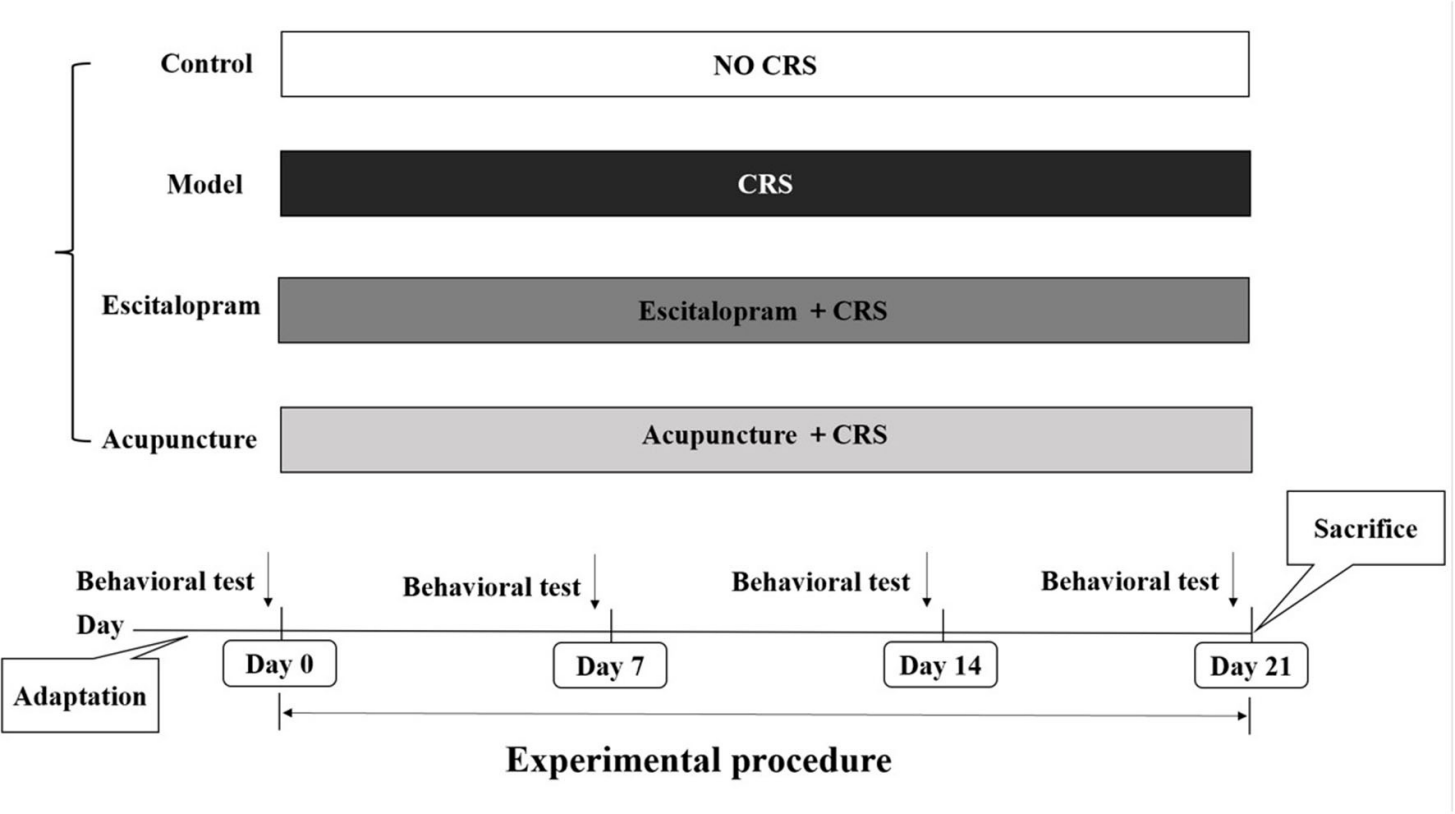
Experimental procedures. CRS, chronic restraint stress; Behavioral tests, including body weight assessment and sucrose preference test.

Male Sprague-Dawley (SD) rats, weighting 180 ± 20 g, were included in the present experiment. All animals were obtained from Weitong Lihua Experimental Animal Center of Beijing, China. All protocols were approved by the Animal Ethics Committee, Beijing University of Chinese Medicine, China (Permission number: BUCM-4-2020102801-4048). All rats were housed in the cages with free access to water and food, under the circadian of 12-h light/dark, and the ambient temperature and relative humidity were maintained at 23–26°C and 50 ± 2%. After 7 days of adaption, body weight assessment and sucrose preference test were conducted, and a total of 44 rats with the same baseline of behavioral assessment were included in this study. Then, they were randomly divided into control group, model group, escitalopram group, and acupuncture group (see Figure 1), with 11 rats in each group.

### Establishment of the Animal Model of Depression

Studies have confirmed that CRS procedures can well simulate the pathological process of depression (34). Accordingly, the establishment of the animal model of depression in this study was conducted to be the validation of CRS referring to the previous study (35–37). Except for rats in the control group, all rats were exposed to CRS and social isolation for 21 days continuously. Rats were restrained in a cylinder-shaped wire net (20.5 cm long and 6.5 cm in diameter), fixing both ends with a 64 mm long butterfly clip from 10 a.m. to 4 p.m. The wire net was soft enough to prevent from body impairment of rats. During CRS procedures, rats were subjected to food and water deprivation. After CRS procedures, all rats were put back into the cage and they had free access to food and water.

### Escitalopram and Acupuncture Intervention

One hour before CRS procedure, rats in escitalopram group were subjected to the suspension of escitalopram and saline administration (30 mg/100 ml) by gavage (3 mg/kg·d) once per day. The escitalopram oxalate tablets were obtained from Lexapro (H. Lundbeck A/S, 2666079). One hour before CRS procedure, rats in acupuncture group were exposed to acupuncture stimulation, 20 min per session, one session daily for 21 days. The acupoints were selected to be Bai hui (GV20, located at the midline of the head) and Yin tang (GV 29, located at the midpoint between the two eyes). The acupuncture needles were obtained from Suzhou Medical Instrument Co., LTD. (0.25 × 13 mm, No.: 213052). When acupuncture intervention was conducted, rats were placed in separate room under the condition of free activities. Additional stress was strictly avoided during the procedure.

### Behavioral Assessment

All observations were conducted under relatively quiet and dark circumstances. Anhedonia and nutritional status were evaluated by sucrose preference test and body weight assessment, respectively.

#### Body Weight Assessment

The changes of body weight at different time points were observed to evaluate the states of food preference and nutrition status. Body weight assessment was detected at 0 day pre-intervention and at 7, 14, 21 days post-intervention for each rat throughout the experimental procedures.

#### Sucrose Preference Test

Anhedonia was usually expressed by reduced sucrose consumption in the animal model of depression (38). Rats were trained to adapt to 1% sucrose solution (Sigma-Aldrich, Lot No.513H051) during the acclimation cycle. Before the sucrose preference test (SPT), all rats were deprived of food and water for 23 h. Then all rats had free access to two pre-weighed bottles containing 150 ml 1% sucrose solution and 150 ml pure water for 1 h. At the end of the test, the bottles liquid were re-weighed and recorded to calculate the sucrose preference rate. Sucrose preference rate (%) = sucrose consumption / (sucrose consumption + water consumption) × 100% (35, 36, 38).

### Tissue Collection and Processing Procedures

After 21 days of experiment cycle, samples collection were conducted on day 22. No rats died during the experiment. Five rats of each group were anesthetized with 10% chloral hydrate and perfused transcardially with 0.9% saline followed by 4% paraformaldehyde solution. The intact brain was removed after perfusion and fixed in 4% paraformaldehyde solution for the next experimental cycle. After anesthesia, another 6 rats were exposed to blood samples collection taken from the abdominal aorta. After storing at room temperature for 2 h, the blood samples were centrifuged at 3,000 rpm for 10 min (4°C, with a centrifugal radius of 6.6 cm) to separate serum. After the blood samples collection, the hippocampus were isolated from brain on ice quickly and put into cryopreservation tubes, which were quickly placed in liquid nitrogen. Then the serum samples and hippocampus samples were transferred to−80°C for storage for the next experimental process.

### Immunofluorescence Staining

For the immunofluorescence staining, brain samples were quickly harvested and fixed in 4% PFA solution, then dehydrated in 10% sucrose solution. Later, the brain samples were embedded in OCT (Tissue-Tek) and serial sectioned with cryostat. The brain samples were cut into 10 μm thickness for the following experimental cycle. Staining was performed on the hippocampal sections fixed on slide glass, air dried for 20 min at room temperature. For permeabilization, slides were fixed with cold acetone for 7 min. Non-specific binding was blocked with 4% donkey serum for 1 h. The slides were washed with 1 × TBS containing 0.1% Tween-20 and then exposed overnight to the following primary antibody mixtures: IBA1 Rabbit Polyclonal antibody (1:500, Proteintech, 10904-1-AP, USA), Anti-HMGB1 antibody - N-terminal (1:500, Abcam, ab228624, USA) at 4°C. Detection of primary antibodies was performed with secondary antibody [Goat Anti-Rabbit IgG H&L (HRP) (1:1000, Abcam, ab6721, USA] for 2 h in the darkness. The sections were then washed five times with PBS. Detection of nucleus was performed with DAPI staining solution (1:1000, Abcam, ab228549, USA) and then incubated at room temperature for 5 min. The slides were washed with PBS for 3 times, air dried for 30 min at room temperature. Finally, the anti-fluorescent extractant was added. Immunofluorescence sections were observed. And the morphology and expression of microglia and HMGB1 were recorded with fluorescence microscope, using excitation wavelengths of 633 nm (helium/neon2, blue Cy5 labeling), 543 nm (helium/neon1, red Cy3 immunofluorescence). Images were captured using a Case Viewer software and analyzed using Fiji (ImageJ) by a condition-blind observer.

### Western Blotting

BCA assay was used to determine the protein concentration. Then the sample of hippocampus were homogenized in RIPA buffer, and the final mass concentration of the sample was 4 g/L, the loading amount of the protein sample to be tested was 160 μL. The final mass concentration of 3 samples from separate groups was <4 g/L. Accordingly, 5 samples from each group were used to the detection of western blotting. Protein samples were run on 12% Tris-glycine SDS-PAGE gels, transferred to polyvinylidene difluoride (PVDF) membrane (0.45 μm, Millipore, Massachusetts, USA), and blotted with antibodies against IL-10 (1:1000, Abcam), GAPDH (1:5000, Abcam). Primary antibody incubation was performed overnight at 4 °C. Secondary antibodies (1:1000, Abcam) were incubated for 40 min at room temperature. After the exposure film is scanned, the image is analyzed by Gel Image Ver 4.0 software and the band levels were quantified. The result is the gray value of the target strip/ GAPDH gray value represents the correction error.

### ELISA

After the blood samples collection, 2 samples of the blood from separate groups showed hemolysis. Accordingly, 5 samples from each group were used to the detection of ELISA. The content of serum TNF-α was determined by enzyme-linked immunosorbent assay (ELISA). TNF-α ELISA kits (Ray Biotech. Inc., lot: 0611210709, USA) was used according to the manufacturer's protocol. The photometric measurements were performed at 450 nm according to the instruction of the manufacture.

### Statistical Analysis

The statistical analysis was performed by SPSS 20.0 (IBM, New York, USA). The results of behavioral tests are expressed as mean ± standard deviation (SD), and analyzed by repeated measures analysis of variance. Data were tested for normal distribution and homogeneity of variance. One-way analysis of variance (ANOVA) with Least-Significant Difference (LSD) *post-hoc* test was performed for assessing between-group differences. Non-parametric test was used if variance is not uniform or does not conform to normal distribution. A level of *P* < 0.05 was considered to be significant for analysis.

## Results

### Chronic Restraint Stress Induces Depressive-Like Behavior in Rats

#### Effect of Acupuncture on the Changes of Body Weight of CRS Rats

**Figure 2:**
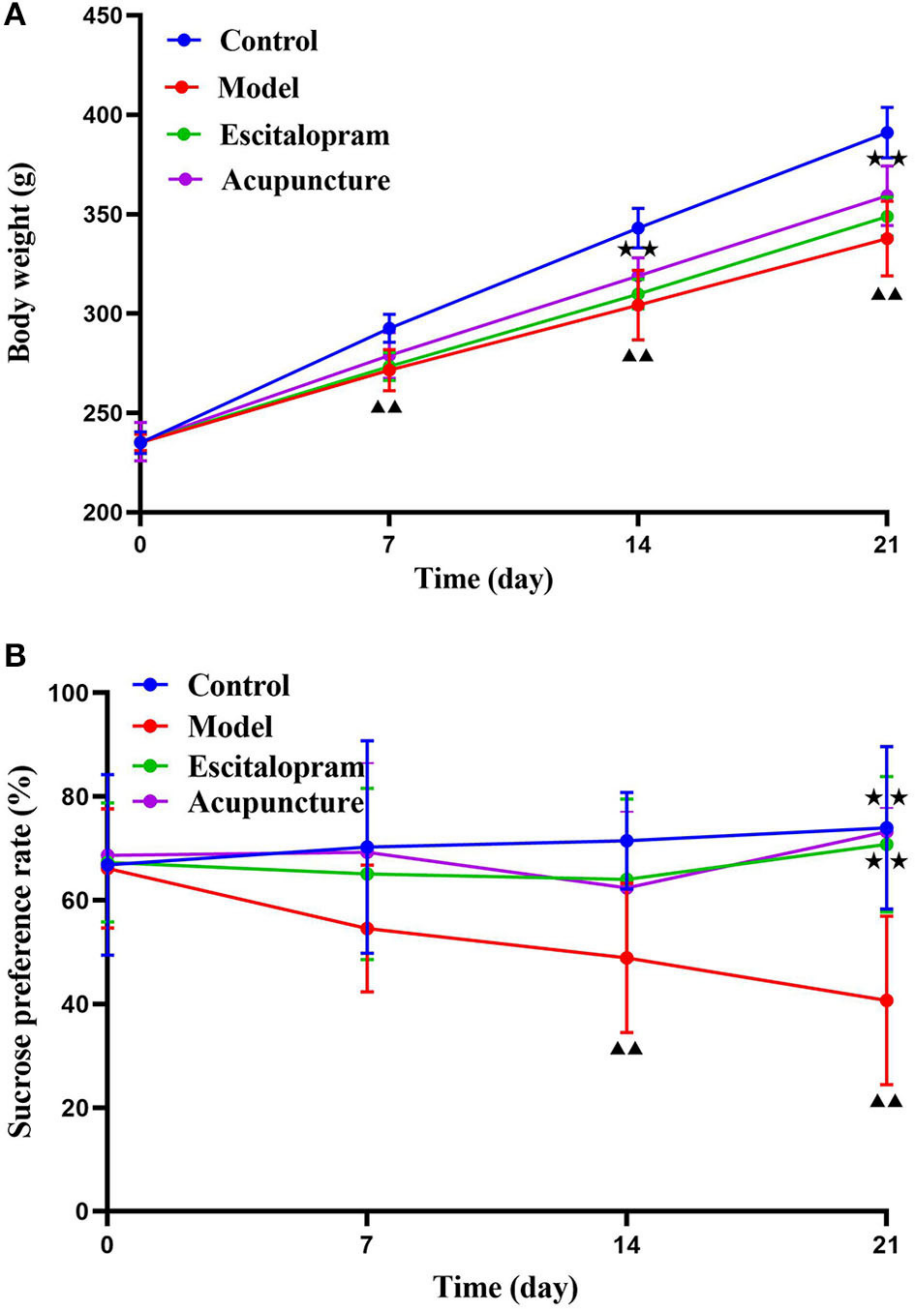
Effects of acupuncture on the CRS-induced depressive-like behaviors in rats. **(A)** Effect of acupuncture on the changes of body weight of CRS rats; ^▴▴^*P* < 0.01, compared with control group; ^⋆⋆^*P* < 0.05, compared with model group. **(B)** Effect of acupuncture on the changes of sucrose preference rate of CRS rats. ^▴▴^*P* < 0.01, compared with control group; ^⋆⋆^*P* < 0.01, compared with model group. Repeated ANOVA followed by LSD's *post-hoc* test.

As shown in Figure 2A and Table 1, there was no significance of the body weight of each group before the experiment. After CRS procedures, the body weight of rats in different groups changed (*F* = 4.488, *P* = 0.000). Compared with the control group, the body weight of the rats in the model group reduced significantly after 7 days of CRS procedures, even lower than that in the control group significantly until at day 21 (all *P* < 0.01). Compared with the model group, the body weight of the acupuncture group began to increase significantly on the 14th day, and its mean weight on the 14th and 21st days were significantly higher than that in the model group (all *P* < 0.05).

**Table 1:** Effects of acupuncture on the changes of body weight of CRS rats.

**Groups**	**0 day**	**7 days**	**14 days**	**21 days**
Control	235.018 ± 5.388	292.609 ± 6.992	342.945 ± 9.944	391.082 ± 12.769
Model	235.191 ± 4.151	271.545 ± 10.367^▴▴^	304.200 ± 17.528^▴▴^	337.708 ± 18.786^▴▴^
Escitalopram	235.264 ± 4.479	273.409 ± 7.100	309.827 ± 7.455	348.882 ± 9.849
Acupuncture	235.591 ± 9.761	278.939 ± 11.577	318.855 ± 9.225^⋆⋆^	359.259 ± 14.926^⋆⋆^

*(n = 11; $\overset{-}{\chi }$ s; g). Data were expressed as mean ± SD*.

▴▴ *P < 0.01, compared with control group;*

⋆⋆ *P < 0.05, compared with model group*.

#### Effects of Acupuncture on the Changes of Sucrose Preference Rate of CRS Rats

As shown in Figure 2B and Table 2, the sucrose preference rate among each group changed with the change of time points after CRS modeling (*F* = 2.642, *P* = 0.008). Compared with the control group, the mean value of sucrose preference rate in the model group decreased significantly at 14 days and 21 days (all *P* < 0.01), which indicated the stress-induced anhedonia and depressive-like behavior. Compared with the model group, acupuncture and escitalopram intervention had a reversal effect on the 21st day, and the sucrose preference index increased significantly (all *P* < 0.01), suggesting that acupuncture can significantly alleviate the CRS-induced anhedonia.

**Table 2:** Effects of acupuncture on the changes of sucrose preference rate of CRS rats.

**Groups**	**0 day**	**7 days**	**14 days**	**21 days**
Control	66.836 ± 17.435	70.273 ± 20.504	71.518 ± 9.349	73.991 ± 15.693
Model	66.136 ± 11.473	54.545 ± 12.212	48.873 ± 14.421^▴▴^	40.709 ± 16.256^▴▴^
Escitalopram	67.309 ± 11.497	65.091 ± 16.514	64.027 ± 15.527	70.782 ± 13.106^⋆⋆^
Acupuncture	68.691 ± 9.155	69.255 ± 17.230	62.382 ± 14.745	73.264 ± 4.561^⋆⋆^

*(n = 11; $\overset{-}{\chi }$ ± s; %). Data were expressed as mean ± SD*.

▴▴ *P < 0.01, compared with control group;*

⋆⋆ *P < 0.01, compared with model group*.

### Effects of Acupuncture on the Expression of HMGB1 and IBA-1 in the Hippocampus of CRS Rats

**Figure 3:**
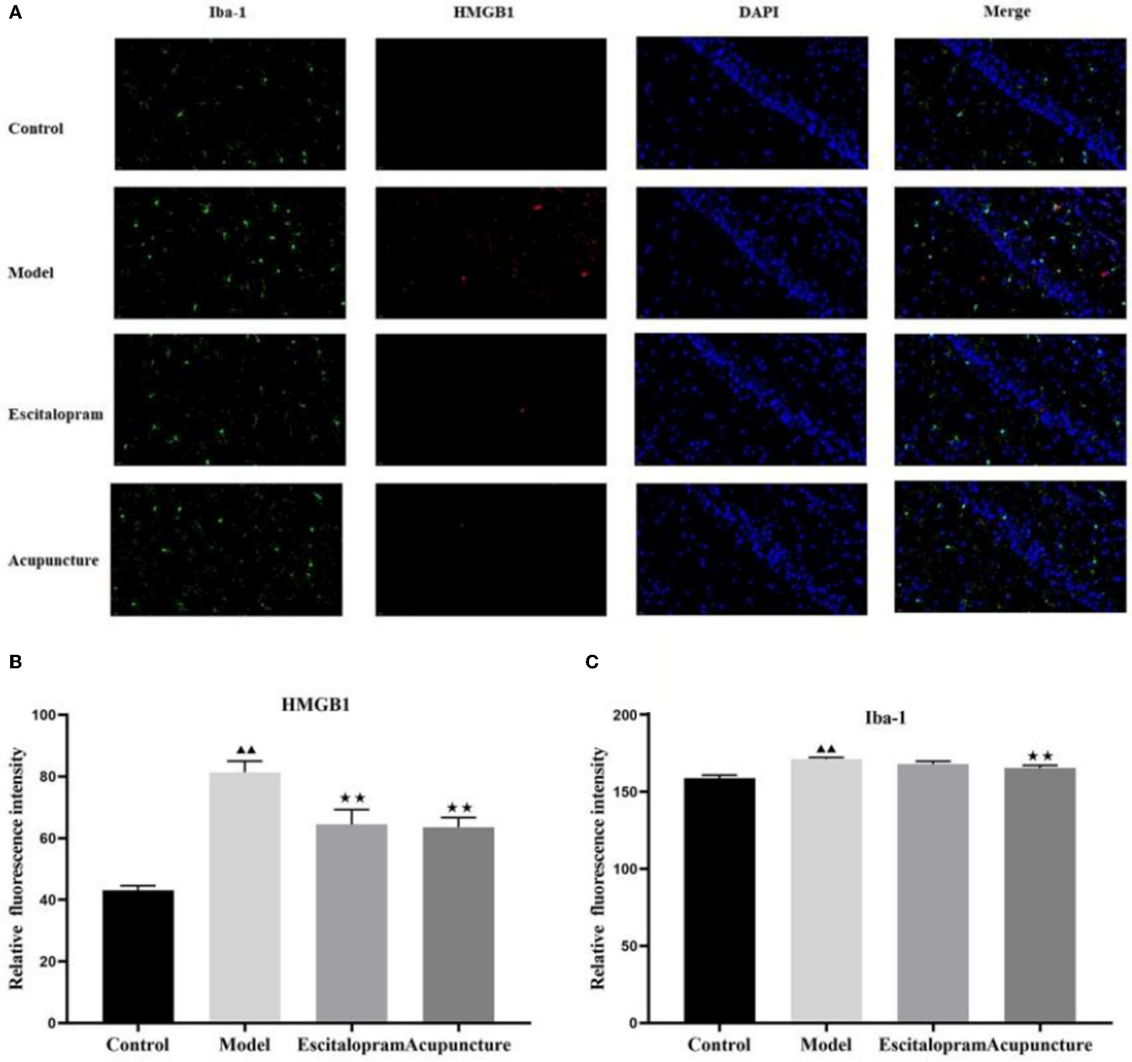
Effect of acupuncture on the expression of HMGB1 and IBA-1 in the hippocampus of CRS rats. **(A)** Immunofluorescence staining of hippocampal CA1 region in different groups (× 40). IBA-1, green; HMGB1, red; DAPI, blue; scale bar, 20 μm; **(B)** Relative fluorescence intensity of HMGB1; **(C)** Relative fluorescence intensity of IBA-1. Data was expressed as means ± SEM (n = 5). ^▴▴^*P* < 0.01, compared with control group; ^⋆⋆^*P* < 0.05, compared with model group. One-way ANOVA followed by LSD's *post-hoc* test.

As illustrated in Figures 3A–C and Table 3, following 21 days of intervention, there was a significant difference in the expression of HMGB1 (*F* = 11.863, *P* < 0.01) and IBA-1 (*F* = 9.106, *P* < 0.01) in the hippocampus CA1 region among groups. The expression of HMGB1 and IBA-1 in the hippocampus of the model group was significantly higher than that of the control group (all *P* < 0.01). Compared with the model group, acupuncture and escitalopram intervention reversed the increase of HMGB1 (all *P* < 0.05), and acupuncture treatment had down-regulated the expression of IBA-1 in the hippocampus CA1 region of CRS rats (Figures 3A–C; Table 3).

**Table 3:** Effects of acupuncture on the expression of HMGB1 and IBA-1 in the hippocampus of CRS rats.

**Groups**	**HMGB1 relative fluorescence intensity**	**IBA-1 relative fluorescence intensity**
Control	43.083 ± 1.478	158.858 ± 1.928
Model	81.343 ± 3.638^▴▴^	171.027 ± 1.199^▴▴^
Escitalopram	64.453 ± 4.810^⋆⋆^	167.955 ± 1.781
Acupuncture	63.573 ± 3.089^⋆⋆^	165.311 ± 1.855^⋆⋆^

*(n = 5; $\overset{-}{\chi }$ ± S.E.). Data are expressed as mean ± SEM*.

▴▴ *P < 0.01, compared with control group;*

⋆⋆ *P < 0.05, compared with model group*.

### Effects of Acupuncture on the Expression of IL-10 Protein in the Hippocampus of CRS Rats

**Figure 4:**
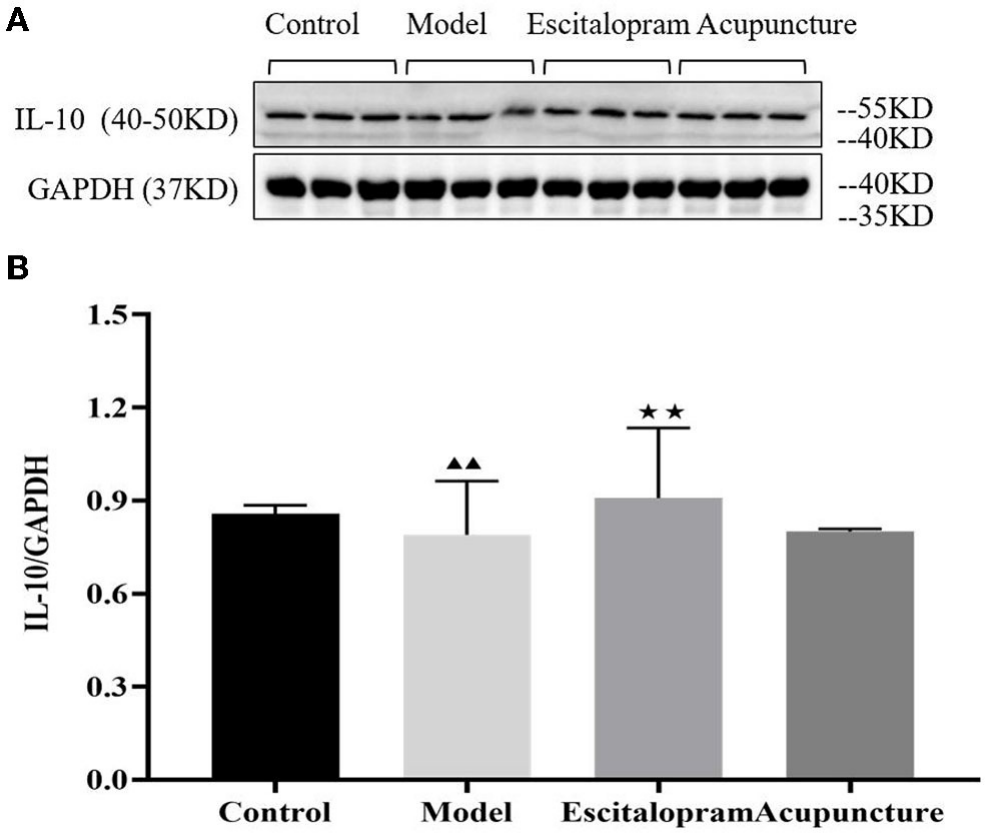
**(A)** is the protein band diagram of IL-10, and **(B)** is the bar diagram of IL-10. Effects of acupuncture on the expression of IL-10 protein in the hippocampus of CRS rats. Data are expressed as means ± S.E.M. (*n* = 5). ^▴▴^*P* < 0.05, compared with control group; ^⋆⋆^*P* < 0.01, compared with model group. One-way ANOVA followed by LSD's *post-hoc* test.

As the results showed in Figures 4A,B and Table 4, compared with the control group, the expression of IL-10 protein in the hippocampus of the model group decreased significantly (*P* < 0.05). Compared with the model group, escitalopram intervention had an obvious upward trend on the expression of IL-10 protein in the hippocampus of CRS rats (*P* < 0.01).

**Table 4:** Effects of acupuncture on the expression of IL-10 protein in the hippocampus of CRS rats.

**Groups**	**IL-10/GAPDH**
Control	0.858 ± 0.027
Model	0.790 ± 0.173^▴▴^
Escitalopram	0.908 ± 0.226^⋆⋆^
Acupuncture	0.801 ± 0.008

*(n = 5; $\overset{-}{\chi }$ ± S.E.). Data are expressed as means ± SEM*.

▴▴ *P < 0.05, compared with control group;*

⋆⋆ *P < 0.01, compared with model group*.

### Effects of Acupuncture on the Content of Serum TNF-α of CRS Rats

**Figure 5:**
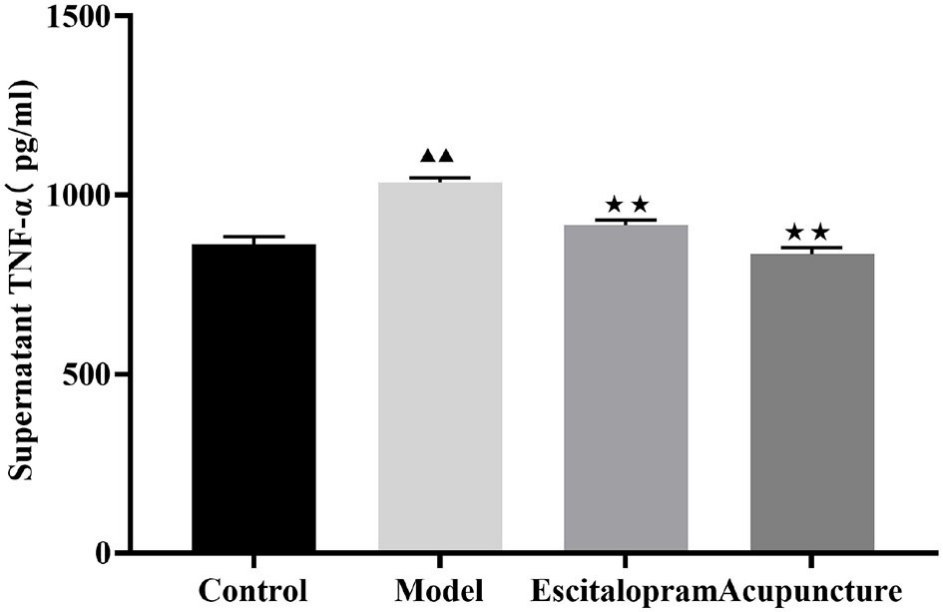
Effects of acupuncture on the content of serum TNF-α of CRS rats. Data are expressed as means ± S.E.M. (*n* = 5). ^▴▴^*P* < 0.01, compared with control group; ^⋆⋆^*P* < 0.01, compared with model group. One-way ANOVA followed by LSD's *post-hoc* test.

As shown in Figure 5 and Table 5, there was significant differences of serum TNF-α among groups (*F* = 27.152, *P* = 0.000). Compared with the control group, serum pro-inflammatory cytokine of TNF-α was significantly increased in the CRS-mediated model group (*P* < 0.01). Compared with the model group, both acupuncture and escitalopram reversed the high expression of TNF-α in serum-induced by CRS rats (*P* < 0.01).

**Table 5:** Effects of acupuncture on the content of serum TNF-α of CRS rats.

**Groups**	**TNF-α (pg/ml)**
Control	862.375 ± 21.292
Model	1034.841 ± 12.874^▴▴^
Escitalopram	915.608 ± 15.009^⋆⋆^
Acupuncture	834.801 ± 17.632^⋆⋆^

*(n = 5; $\overset{-}{\chi }$ ± S.E.). Data are expressed as means ± SEM*.

▴▴ *P < 0.01, compared with control group;*

⋆⋆ *P < 0.01, compared with model group*.

## Discussion

Studies have confirmed that CRS procedures can well simulate the pathological process of depression. Previous studies have verified that CRS could contribute to depressive-like behaviors in the rat model (35, 36). Accordingly, the establishment of the animal model of depression in this study was conducted to be the validation of CRS procedures. We aimed to elucidate the potential mechanisms underlying the antidepressant effect of acupuncture on modulating the neuroinflammation induced by HMGB1. In the present study, we identified the findings on the antidepressant effect of acupuncture from the microglia and cytokines level alteration. The results have shown that CRS procedures obviously induced depression-like behaviors, which might be triggered by the neuroinflammation through the stress-induced up-regulation of the expression of HMGB1 and IBA-1 in the hippocampus. Notably, acupuncture significantly alleviated the depression-like behaviors and reversed the high expression of HMGB1, Iba-1, and TNF-α in CRS rats, which indicates the antidepressant effect of acupuncture and provides new experimental evidence for new therapies in the treatment of depression.

### CRS Induces Depressive-Like Behaviors and Acupuncture Exerts the Antidepressant-Like Effect

In the present study, the changes of behavioral assessment at different time points were observed to evaluate the states of nutrition and anhedonia (39–41). There were consistent baseline values among different groups at 0 day before intervention. After the CRS procedures, a significant comparative difference of behaviors was found. Compared with the control group, the weight of rats was significantly decreased in the model group at 7, 14, and 21 days. Compared with the model group, acupuncture intervention increased the body weight at 14 and 21days, while there was no significant difference from the escitalopram group at four-time points. Our present study manifests that acupuncture has effectively regulated the weight loss of CRS rats and produced positive therapeutic effects. Accordingly, we will focus on relative indicators to elucidate the additional specific mechanism of the significant change in the body weight in our following experiment. Previous studies had found that anhedonia was evaluated the depression-like behavior in rats by detecting the SPT. Compared with the control group, we found that exposure to CRS induced depressive-like behavior in the SPT, which mainly showed reduced sucrose preference rate at 14, 21 days. Acupuncture and escitalopram intervention reversed the decrease of sucrose preference rate at 21 days, which suggested that acupuncture alleviated the CRS-induced depressive-like behaviors.

Acupuncture is used to regulate painful diseases or psychological states, including headaches, arthritis, depression, or anxiety (42). Acupuncture, a traditional Chinese medicine treatment method, has the effect of anti-depression and reducing the severity of depressive symptoms (43). According to the findings from the Resting-State fMRI, acupuncture can induce different brain activity at different points. It has been reported that acupuncture at combined acupoints could activate a wider range of brain areas than single acupoint, and the areas regulated by acupuncture are mostly related to emotion and cognition (44). Previous studies have also shown that acupuncture at *Bai hui* (GV 20) and *Yin tang* (GV 29) can regulate the hippocampal injury, and alleviate the depression state of rats and exert antidepressant effects (35). However, single acupuncture at either *Bai hui* or *Yin tang* fails to alleviate the state of depression (45). Therefore, the acupoints of *Bai hui* (GV 20) and *Yin tang* (GV 29) were selected to be investigated the antidepressant mechanisms of acupuncture. The results of the present study have identified that CRS induced depressive-like behaviors and acupuncture at *Bai hui* (GV 20) and *Yin tang* (GV 29) exhibited the antidepressant-like effect.

### Acupuncture Reversed CRS-Induced Neuroinflammation Mediated by Hippocampal Iba-1 and HMGB1

Evidence is increasing that psychological and physical stressors could activate immune and inflammation processes, contributing to depressive symptoms. As is common knowledge, the activation of microglia is a specific manifestation of the neuroinflammation of the central nervous system (CNS) mediated by stress (2, 12, 14). The activation of HMGB1 could promote depressive-like behaviors in stress models of depression (22, 23). HMGB1 has been considered to be the mediator to initiate the microglia sensitization and activation of proinflammatory cytokines (20, 27, 28). Ionized calcium binding adaptor molecule 1 (Iba-1) is considered to be the microglia marker. The changes of the concentration of Iba-1 and HMGB1 in hippocampus were considered to be involved in the pathological process of depression. The hippocampus, the regulatory center of the hypothalamic-pituitary-adrenal (HPA) axis, is considered to be involved in the pathological process of depression (46, 47). It has suggested that stress increases the number of microglia in the CA1 and CA3 region of the hippocampus (48), and increases the Iba-1 level (49), significantly increasing relative protein level and fluorescence density of Iba-1 in the hippocampus of CA1 region (50). HMGB1 could induce systemic inflammatory reactions, such as cold, arthritis, anorexia, or weight loss (51). It has also been identified the significantly upregulated expression of HMGB1 in the serum and cerebral cortex of chronic unpredictable mild stress (CUMS)-induced depression mouse model (26). CUMS stress caused concentrations of HMGB1 substantially increased in the hippocampus and serum (52). HMGB1 is involved in depression-like behavior induced by lipopolysaccharide, and mice were used with human Recombinant HMGB1 (rHMGB1) to produce depression-like behavior (23). In our present study, the result indicated that CRS induced the dramatic activation of microglia and up-regulation of HMGB1, which was in accordance with the previous studies indicating the stress-induced neuroinflammation mediated by hippocampal Iba-1 and HMGB1 (53–56). Importantly, Acupuncture intervention reversed the CRS-induced increase of HMGB1 and Iba-1 in the hippocampus of CRS rats, suggesting that acupuncture exhibited the antidepressant effect by regulating the changes of hippocampal microglia and HMGB1 levels, which is consistent with our theoretical hypothesis. At the same time, our results confirmed that it can produce antidepressant effects through the regulation of microglia and HMGB1, which is consistent with the previous results (48–50).

Studies have indicated that hyperactivation of microglia can induce depression by secreting proinflammatory factors (15, 16). The patients suffered from depression generally exhibited an elevated amount of proinflammatory cytokines in the serum, microglia activation, and neuronal deficit in the CNS (54). Depression in the absence of other diseases has been shown to be associated with increased levels of various pro-inflammatory cytokines, including TNF-α and interleukin (57). Data from the clinical study indicated that MDD patients exhibited increased levels of serum IL-1β and TNF-α (58). The animal studies have also identified that stress increased the levels of hippocampal inflammation factors, including IL-1β and TNF-α (49, 50). TNF-α induces similarly depression-like symptoms in mice (6). However, IL-10 can reduce the harmful effects of cytokines on memory and plasticity. Studies have shown that administration of IL-10 can rescue depression-related learning and memory defects and restore the density of hippocampal dendritic spines (49). In the present study, our results showed that rats exposed to CRS exerted decrease of IL-10 in the hippocampus while increase of TNF-α in serum, which is consistent with the reported studies (59, 60). However, it is worth noting that both acupuncture and escitalopram intervention could down-regulate the expression of serum TNF-α, and the escitalopram administration intervention can increase the expression of IL-10 in the hippocampus, indicating that acupuncture and could regulate the CRS-induced proinflammatory factor TNF-α and exhibited antidepressant effect.

## Conclusion

Conclusively, all of these results give more support to the hypothesis that the CRS-induced neuroinflammation mediated by hippocampal Iba-1 and HMGB1 was involved in the pathogenesis of depression, and the antidepressant effect of acupuncture might be through modulating the neuroinflammation mediated by hippocampal Iba-1 and HMGB1. Notably, the current study preliminarily highlights the role of acupuncture in alleviating depressive behavior associated with stress-induced neuroinflammation mediated by HMGB1 in the CRS model of depression, which might provide new experimental evidence for new therapies in the treatment of depression. However, there are several limitations of the present study. No specific blocker of HMGB1 was applied to identify whether acupuncture has specificity in the regulation of HMGB1. Meanwhile, our previous studies from clinical investigation has identified the therapeutic effect of acupuncture in the treatment of depression. Accordingly, sham acupuncture was not used in our study. In the future, our team will continue to carry out the study of the antidepressant effect of acupuncture based on the present findings.

## Data Availability Statement

The original contributions presented in the study are included in the article. Further inquiries can be directed to the corresponding author.

## Ethics Statement

The animal study was reviewed and approved by Animal Ethics Committee, Beijing University of Chinese Medicine, China (Permission number: BUCM-4-2020102801-4048).

## Author Contributions

HJ obtained the funding and designed the research. TB, XR, and HM directed the experiment. LC, YW, SQi, YS, PL, SQu, and WL performed research. LC and HJ analyzed data. HJ and LC wrote the paper and contributed equally to this work.

## Funding

This research was supported by grants from the National Natural Science Foundation of China (81904313 and 81973937) and Key Research Program of Beijing University of Chinese Medicine of China (2020-JYB-ZDGG-060).

## Conflict of Interest

The authors declare that the research was conducted in the absence of any commercial or financial relationships that could be construed as a potential conflict of interest.

## Publisher's Note

All claims expressed in this article are solely those of the authors and do not necessarily represent those of their affiliated organizations, or those of the publisher, the editors and the reviewers. Any product that may be evaluated in this article, or claim that may be made by its manufacturer, is not guaranteed or endorsed by the publisher.
