# Supplementary figures and images for: Acupuncture Ameliorates Depressive Behaviors by Modulating the Expression of Hippocampal Iba-1 and HMGB1 in Rats Exposed to Chronic Restraint Stress
Source: Front Psychiatry. 2022 Jun 6;13:903004. doi: 10.3389/fpsyt.2022.903004 (PMC9207245; doi:10.3389/fpsyt.2022.903004)

## Slide 1
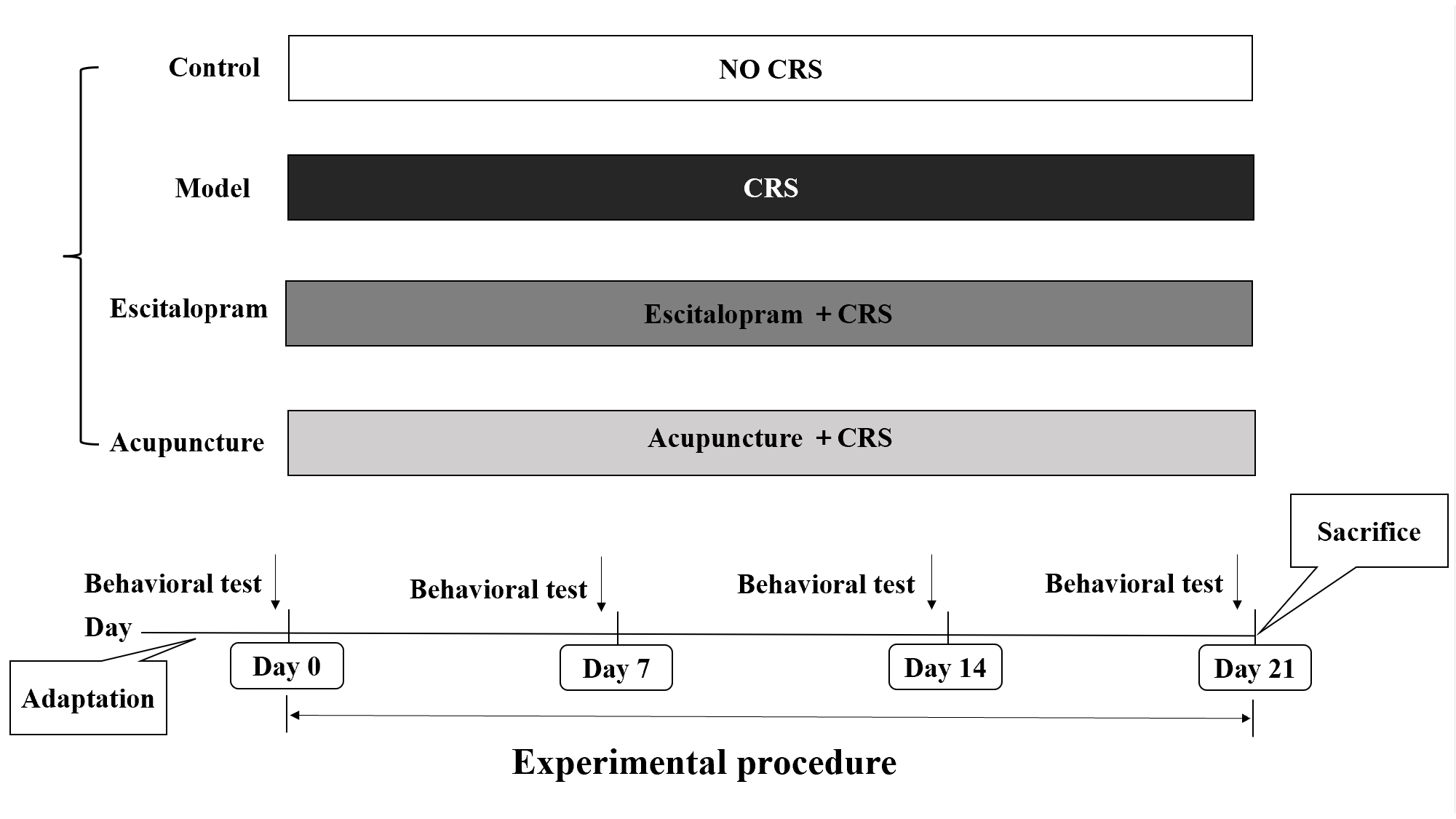

Supplement: Supplementary file 1 [file Presentation_1.PPTX]

## Slide 1
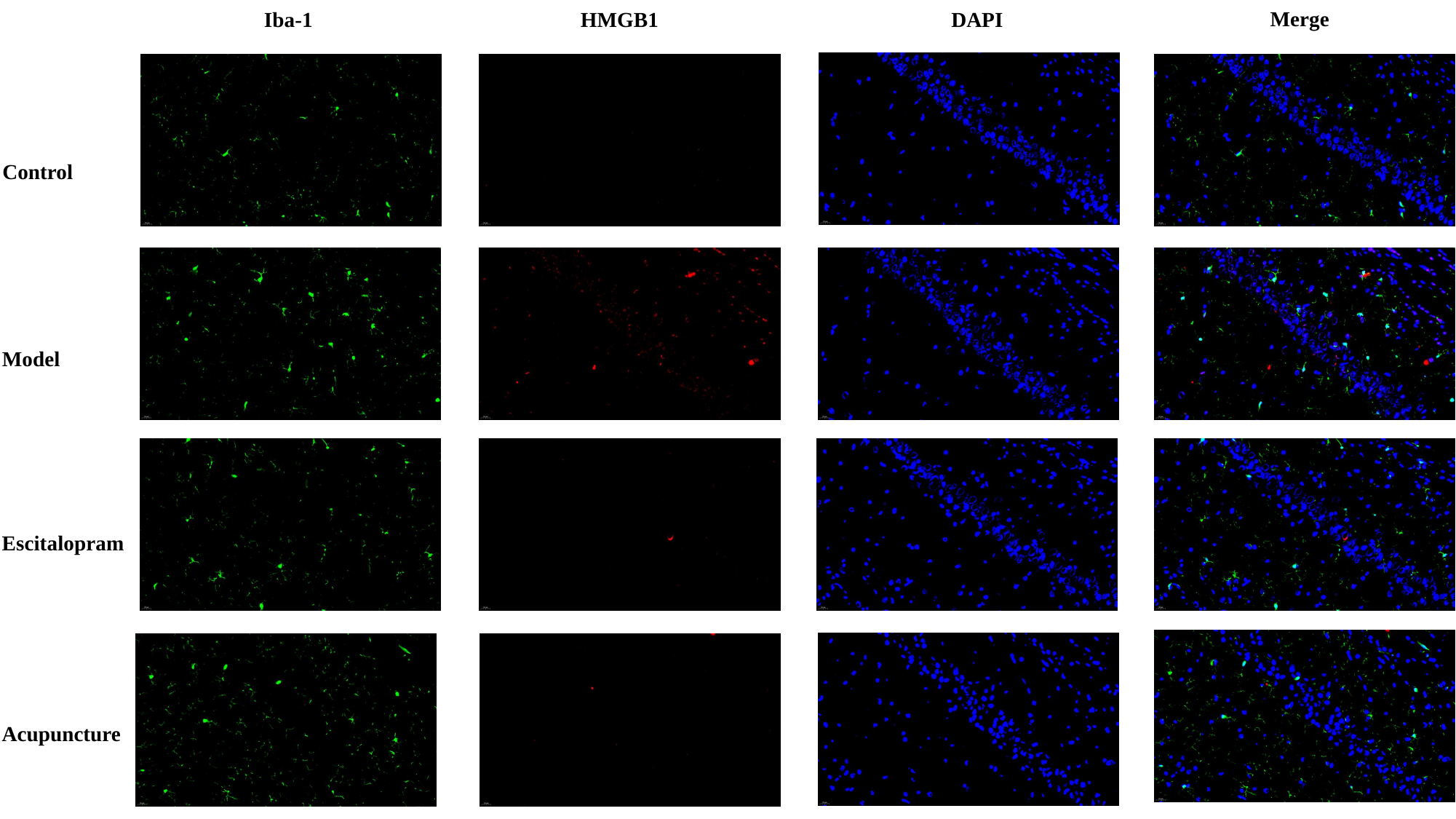

Merge
Iba-1
HMGB1
DAPI
Control
Model
Escitalopram
Acupuncture

Supplement: Supplementary file 2 [file Presentation_2.PPTX]

## Slide 1
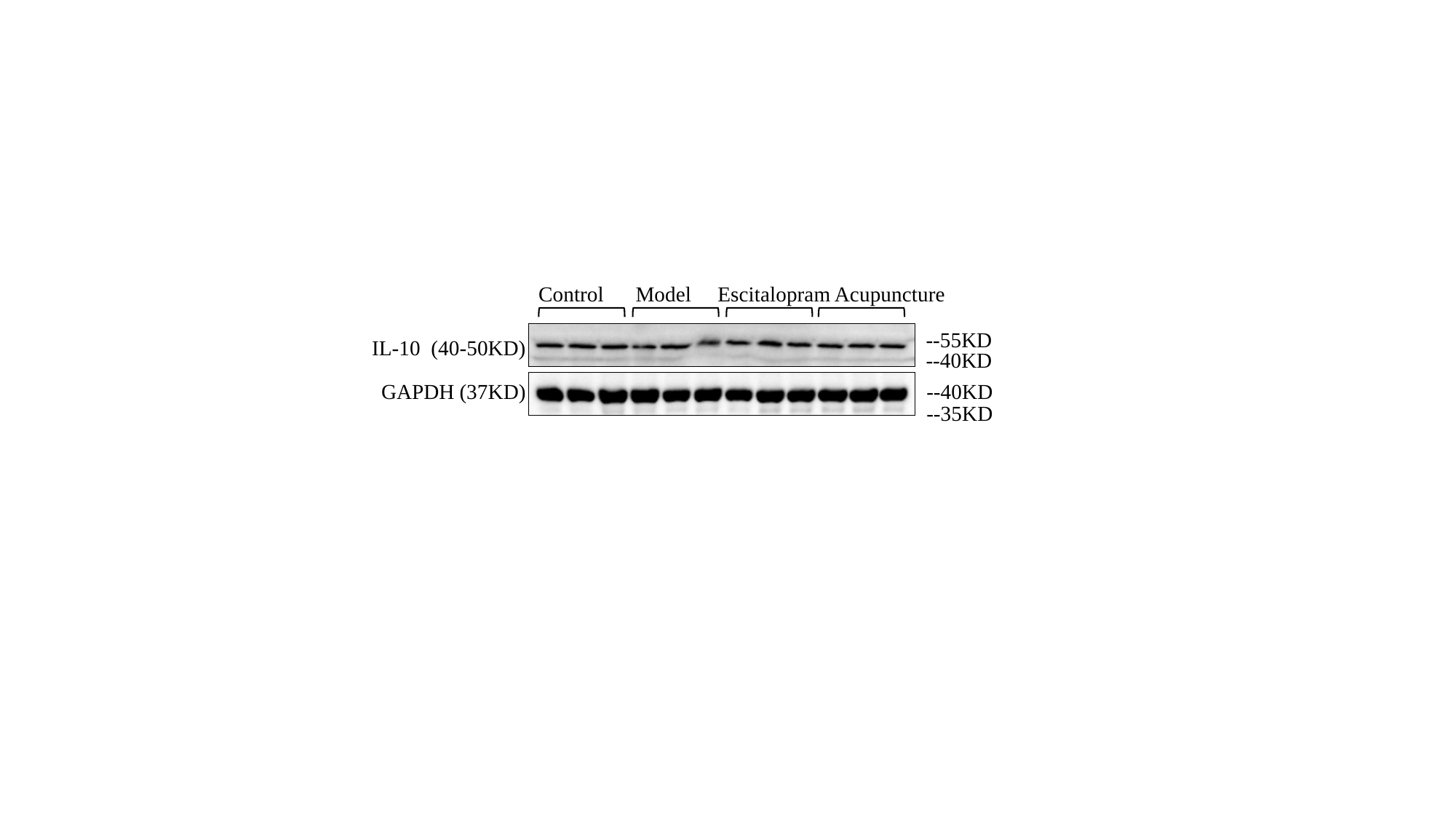

Control Model Escitalopram Acupuncture
--55KD
IL-10 (40-50KD)
--40KD
GAPDH (37KD)
--40KD
--35KD

Supplement: Supplementary file 3 [file Presentation_3.PPTX]

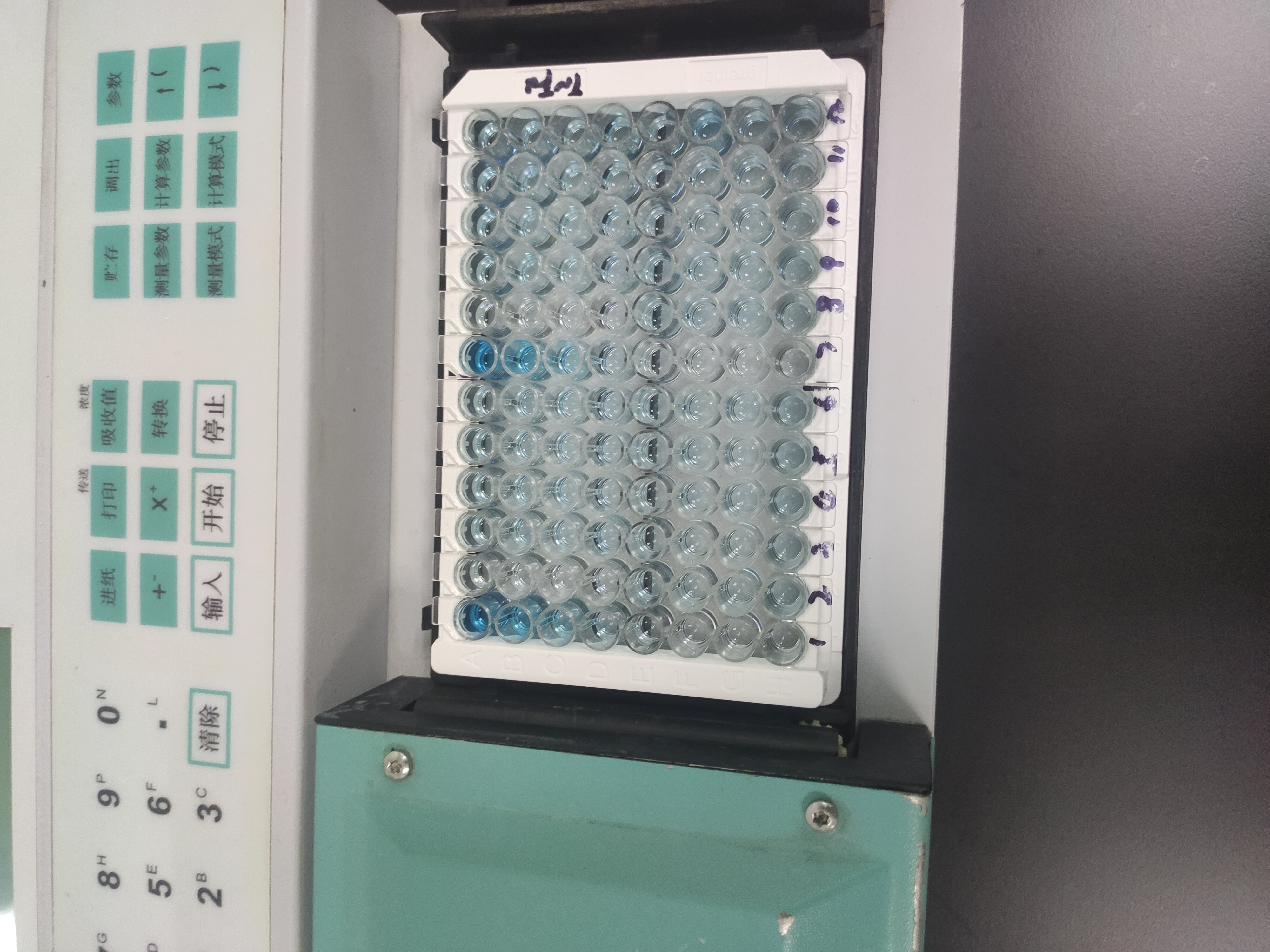

Supplement: Supplementary file 4 [file Data_Sheet_1.ZIP › TNF-a┴ ELISA-original data-Original Data for the results/TMB╧╘╔1⁄2╢┴╓╡.jpg]

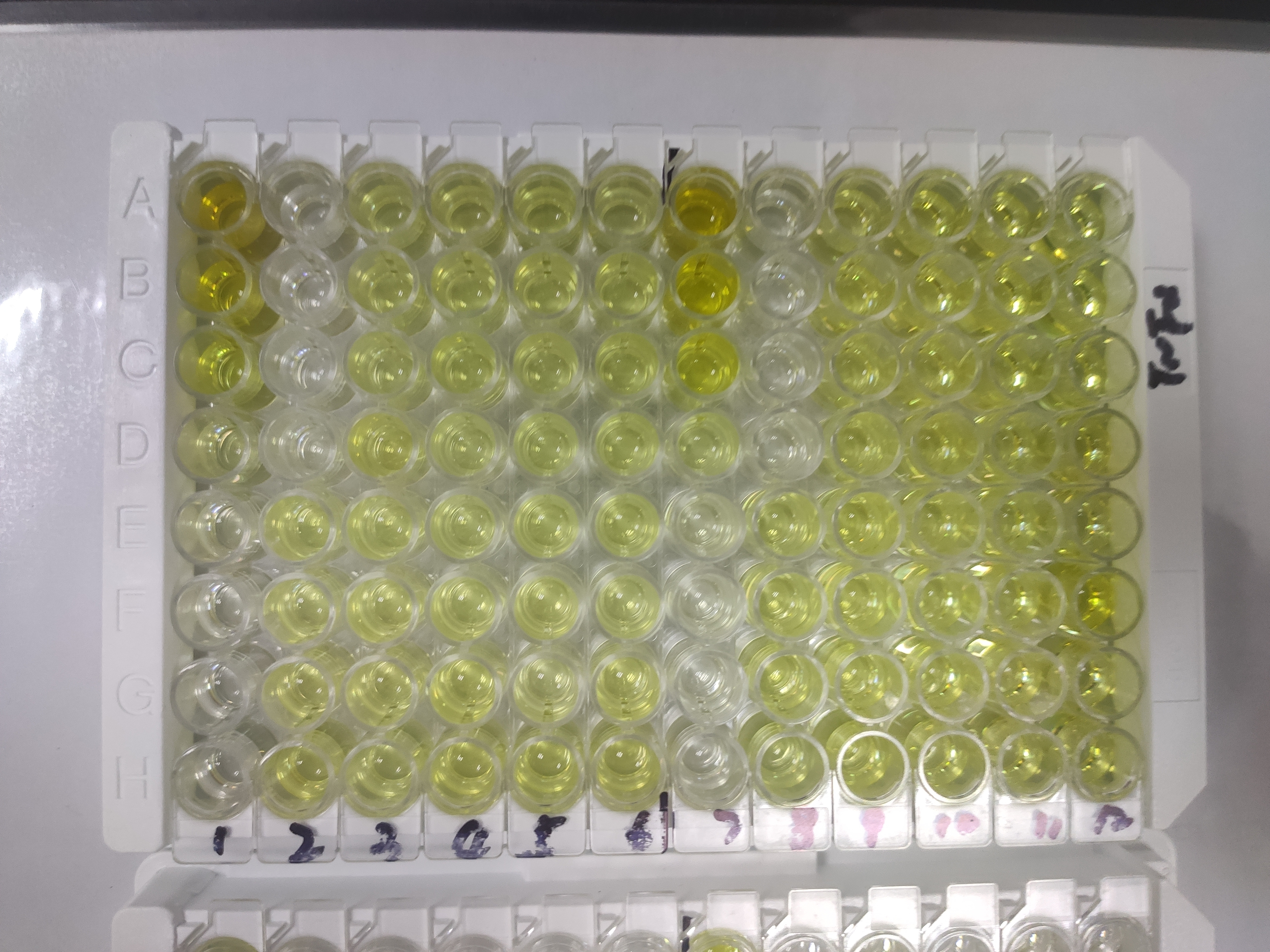

Supplement: Supplementary file 4 [file Data_Sheet_1.ZIP › TNF-a┴ ELISA-original data-Original Data for the results/TNFa╢┴░σ╒╒╞1⁄4 (2).jpg]
